# Characteristics of vaginal microbiota in pregnant women with cervical insufficiency and its association with adverse pregnancy outcomes

**DOI:** 10.12669/pjms.41.5.11966

**Published:** 2025-05

**Authors:** Xiaoxing Zhang, Yun Chen, Kai Zhu, Xieyan Yu, Jiejing Zhao

**Affiliations:** 1 Xiaoxing Zhang, Department of Gynecology and Obstetrics, Huzhou Maternity & Child health Care Hospital, Huzhou, Zhejiang Province 313000, P.R. China; 2 Yun Chen, Department of Gynecology and Obstetrics, Huzhou Maternity & Child health Care Hospital, Huzhou, Zhejiang Province 313000, P.R. China; 3 Kai Zhu, Department of Pathology, Huzhou Maternity & Child health Care Hospital, Huzhou, Zhejiang Province 313000, P.R. China; 4 Xieyan Yu, Department of Gynecology and Obstetrics, Huzhou Maternity & Child health Care Hospital, Huzhou, Zhejiang Province 313000, P.R. China; 5 Jiejing Zhao, Department of Gynecology and Obstetrics, Huzhou Maternity & Child health Care Hospital, Huzhou, Zhejiang Province 313000, P.R. China

**Keywords:** Adverse pregnancy outcomes, Cervical incompetence, Distribution characteristics, Pregnant women, Vaginal microbiota

## Abstract

**Objective:**

To explore specific characteristics of the vaginal microbiota of pregnant women with cervical insufficiency (CIC) and its association with adverse pregnancy outcomes.

**Methods:**

Records of 337 pregnant women who visited Huzhou Maternity & Child Health Care Hospital between June 2020 and June 2023 were retrospectively analyzed. Based on the diagnosis, the patients were divided into the CIC (n = 225) and control groups (n = 112). The distribution characteristics of vaginal microbiota in pregnant women and its association with adverse pregnancy outcomes were analyzed.

**Results:**

One hundred and nine patients in the CIC group showed significantly higher bacterial positivity than the 36 patients in the control group (*P*<0.05). Bacterial identification results showed a significantly higher number of Ureaplasma urealyticum strains and a considerably lower number of Escherichia coli strains in the CIC group. Among the 225 patients with CIC, 43 had poor pregnancy outcomes. The proportions of Candida albicans, Gardnerella vaginalis, Ureaplasma urealyticum, and Chlamydia were higher in the poor group than in the good group (P < 0.05). The rates of conservative treatment in the group with poor pregnancy outcomes were higher than in the group with good pregnancy outcomes (*P*<0.05). Logistic analysis showed that Candida albicans, Gardnerella vaginalis, Ureaplasma urealyticum, and Chlamydia were risk factors for adverse pregnancy outcomes in patients with CIC (*P*<0.05).

**Conclusions:**

The vaginal microbiota of pregnant women with CIC is diverse, with predominantly Ureaplasma urealyticum. Candida albicans, Gardnerella vaginalis, Ureaplasma urealyticum, and Chlamydia are risk factors for adverse pregnancy outcomes in women with CIC.

## INTRODUCTION

Cervical incompetence (CIC) is a common pregnancy complication characterized by premature dilation of the cervix and/or premature rupture of membranes, leading to premature birth or miscarriage. The mechanism of occurrence may be related to abnormal anatomical structure and function of the cervix.^1^,^2^ At present, cervical cerclage is often used from a surgical perspective to improve pregnancy rates and outcomes.^3^ Fang et al. has reported that dysbiosis of the vaginal flora can lead to failure of cervical cerclage surgery.^4^ With the development of microbiology, an increasing number of studies have shown that vaginal microbiota is an important component of the female reproductive system and plays a crucial role in maintaining female reproductive health.^4^,^5^

Studies have shown that there are differences in the cervical vaginal microbiota between healthy pregnant women and pregnant women with CIC.^5^,^6^ Under normal circumstances, the vaginal microbiota remains relatively stable, with lactobacilli as the dominant bacteria in the cervical microbiota and other resident bacteria, including Bacillus subtilis, Prevotella, and Streptococcus digestus.^7^,^8^ In CIC In CIC pregnant women with CIC, the diversity of vaginal microbiota increases, and some microorganisms may promote inflammatory responses. For example, excessive growth of pathogens, such as Escherichia coli and Klebsiella pneumoniae, can lead to an imbalance in the vaginal microbiota, which may affect cervical function and increase the risk of adverse pregnancy outcomes.^4^,^5^,^9^ In addition, microbial communities can participate in the development of CIC by influencing the secretion of immune-related molecules, such as cytokines and growth factors, or by influencing cellular biological processes, such as apoptosis and autophagy.^10^-^12^

Therefore, we need to conduct in-depth research on the changes in the cervical vaginal microbiota in pregnant women with CIC and explore the factors affecting adverse pregnancy outcomes. This will help us to better understand the pathogenesis of CIC and provide new ideas and methods for the prevention and treatment of CIC. Our hypothesis posited that certain vaginal microbiota are associated with adverse pregnancy outcomes.

## METHODS

This retrospective cohort study analyzed the clinical data of 396 pregnant women who visited Huzhou Maternity & Child Health Care Hospital from June 2020 to June 2023.

### Inclusion criteria:

- Age ≥ 18 years.
- Singleton pregnancy.
- Cervical length ≤ 25 mm at mid-pregnancy transvaginal ultrasound examination.
- Cervical dilation detected during the mid-pregnancy physical examination.
- Patients with complete clinical data.

### Exclusion criteria:

- Patients with a history of diabetes and nephritis before pregnancy.
- Patients with fetal malformation.
- Patients with psychiatric disorders.
- Patients with vital organ dysfunction.
- Patients with a history of hypertension or other cardiovascular diseases before pregnancy.
- Patients with combined malignant tumors.
- Patients with autoimmune deficiency or reproductive organ malformations.
- Patients with other pregnancy complications or comorbidities.
- Patients with abnormal coagulation function and a history of smoking and drinking.

**Fig.1:**
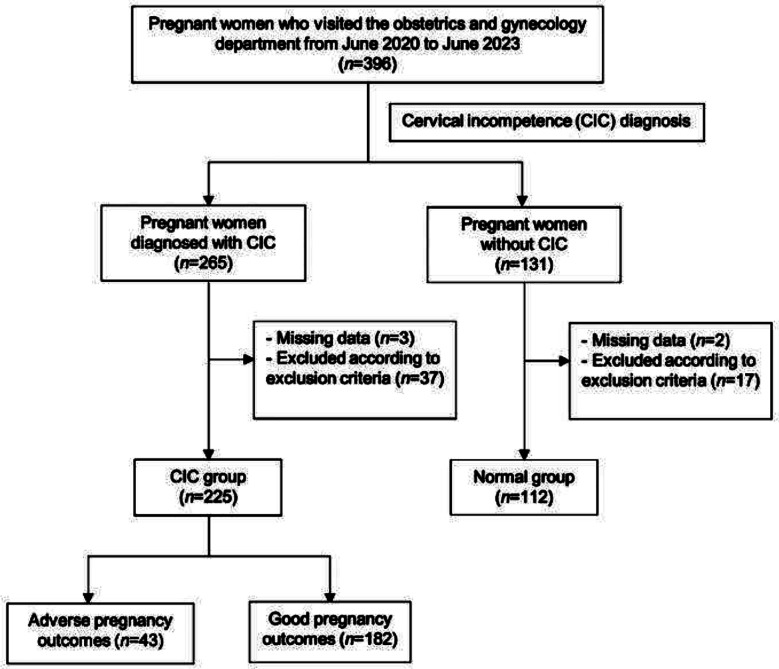
Patient screening flowchart.

The patient screening flowchart is provided in Fig.1. Diagnosis of CIC was made based on the: 1) A history of painless late miscarriage or extremely premature birth with no signs of labor for ≥ three times; 2) A history of painless late miscarriage or extremely premature birth with no signs of delivery ≤ two times, accompanied by one of the following conditions: (i) Cervical length ≤ 25mm measured by transvaginal ultrasound before 24 weeks of pregnancy, accompanied by progressive cervical dilation and cervical canal shortening; (ii) During nonpregnancy, vaginal ultrasound measurement of cervical length ≤ 25mm; (iii) During nonpregnancy, the 8th cervical dilation rod passes through the cervical opening without resistance.

Based on the diagnosis, patients who met the eligibility criteria were retrospectively divided into the CIC and the control group. All study procedures involving human participants adhered to the ethical standards of the institutional and/or national research committee(s) and the Declaration of Helsinki (as revised in 2013). Written informed consent was obtained from patients or legal guardians.

### Ethical Approval:

The Medical Ethics Committee of Huzhou Maternity and Child Health Care Hospital approved this study (No.: 2021-R-014, dated November 16, 2021).

### Data collection:

The clinical data of the participants were collected by reviewing electronic and paper-based medical records and included:

- Demographic data and medical history, including age, pre-pregnancy body mass index (BMI), gravidity, parity, marital status, education level, delivery mode, CIC treatment, the weeks of cervical cerclage surgery, gestational diabetes, gestational hypertension, history of cervical disease, and scarred uterus, were collected;
- Data on distribution characteristics of the vaginal microbiota, including Candida (C. albicans, C. parapsilosis, C. tropicalis, and other Candida), Staphylococcus (Staph. aureus, Staph. epidermidis, and other Staphylococcus), Enterobacteriaceae (E.coli, and other Enterobacteriaceae), Gardnerella vaginalis, Ureaplasma urealyticum, and Chlamydia;
- Data on adverse pregnancy outcomes, including miscarriage, stillbirth, premature birth, low-birth-weight infants, macrosomia, congenital abnormalities, stillbirth, and neonatal death.

### Vaginal microflora analysis:

Vaginal microflora samples were collected and cultured as follows: The perineum was exposed, and the vaginal secretions were cleaned with sterile cotton balls by an obstetrician. A disposable cotton swab was inserted into the cervical canal 1cm inside the posterior vaginal fornix, slowly rotated for three cycles, and allowed to stand for ten seconds. Then the vaginal secretions of the patients were collected and placed in sterile test tubes and sent to the bacteriological room for culture. According to the National Clinical Laboratory Operating Procedures, blood agar inoculation, bacterial culture, and Gram staining were carried out. Escherichia coli (ATCC25922) and Staphylococcus aureus (ATCC25923) were used as quality control samples, and the strains were identified using the Christie–Atkins–Munch-Petersen (CAMP) test. Bacterial strains were identified using the polymerase chain reaction (PCR) and a semi-automated microbiological analyzer (MicroScan Autoscan 4, Dade Behring Inc., USA).

### Statistical analysis:

Data were entered into Microsoft Excel and analyzed using SPSS (version 22.0; IBM Corp, Armonk, NY, USA). For the sample size calculation, the “one in ten” rule was applied to determine the sample size in logistic regression, considering a 10% possibility of incomplete records, to achieve a minimum sample size of 176 required for the study. Normally-distributed continuous variables were expressed as mean and standard deviation (SD), and t-tests were used to compare the two groups. Non-normally distributed continuous variables were expressed as medians and interquartiles, and the Mann-Whitney U test was used for comparison between the two groups. For categorical variables, the frequency distribution was expressed as a percentage. The chi-squared test, Yates’ correction, or Fisher’s exact test were used to compare categorical variables between the two groups. A logistic regression model was used to analyze the risk factors for adverse pregnancy outcomes in patients with CIC. A p-value of less than 0.05 indicated that the difference was statistically significant.

## RESULTS

### Patient characteristics:

This retrospective cohort study evaluated the clinical records of 396 pregnant women. Of them, 337 met the eligibility criteria and were included in this study. According to the diagnostic results, 225 patients were classified as CIC and were assigned to the CIC group; 112 cases of normal pregnancies were assigned to the control group. The baseline characteristics of the two groups were comparable (Table-I).

**Table-I:** Baseline characteristics of the participants [M(P25/P75)].

Characteristics	CIC group (n = 225)	Normal group (n = 112)	P
Age (years)	30 (26, 34)	29 (26, 33)	0.825
Pre-pregnancy BMI (kg/m^2^)	22.90 (21.05, 24.35)	22.70 (21.20, 24.08)	0.487
Gravidity (times)	2 (1, 2)	2 (1, 2)	0.085
Parity (times)	1 (1, 2)	1 (1, 2)	0.052

BMI: body mass index.

### CIC-associated changes in vaginal microbiota:

As shown in Table-II, the CIC diagnosis was associated with a significantly higher rate of bacteriological positivity (*P*<0.05): 109 out of 225 patients (48.44%) in the CIC group were identified as bacteriologically positive. In contrast, 36 out of 112 (32.14%) women in the control group were bacteriologically positive. The number of Ureaplasma urealyticum strains in the CIC group was significantly higher, and the number of Escherichia coli strains was considerably lower than that in the control group (P < 0.05).

**Table-II:** Comparison of vaginal microbiota between the CIC and the control groups.

Pathogenic bacteria		CIC group (n = 109)	Control group (n = 36)	P
Candida	Candida albicans	13 (11.93)	2 (5.56)	0.440^a^
	C. parapsilosis	5 (4.59)	1 (2.78)	1.000^a^
	C. tropicalis	3 (2.75)	2 (5.56)	0.785^a^
	Other Candida	2 (1.83)	0 (0.00)	1.000^b^
Staphylococcus	Staphylococcus aureus	2 (1.83)	3 (8.33)	0.185^a^
	S. epidermidis	2 (1.83)	1 (2.78)	1.000^b^
	Other Staphylococcus	2 (1.83)	1 (2.78)	1.000^b^
Enterobacteriaceae	Escherichia coli	15 (13.76)	11 (30.56)	0.023
	Other Enterobacteriaceae	5 (4.59)	5 (13.89)	0.126^a^
Gardneralla vaginalis		5 (4.59)	1 (2.78)	1.000^a^
Ureaplasma urealyticum		50 (45.87)	8 (22.22)	0.012
Chlamydia		5 (4.59)	1 (2.78)	1.000^a^

### Changes in vaginal microbiota and pregnancy outcomes:

Of 225 patients in the CIC group, 43 had poor pregnancy outcomes, and 182 had good pregnancy outcomes. As shown in Table-III, the proportion of women who received conservative treatment was significantly higher in the group with poor pregnancy outcomes compared to the group with good pregnancy outcomes (P<0.05). The two groups showed no statistically significant difference in other baseline data and medical history (P > 0.05). Levels of Candida albicans, Gardnerella vaginalis, Ureaplasma urealyticum, and Chlamydia in patients with poor pregnancy outcomes were considerably higher than in patients with good outcomes (*P*<0.05). There was no intergroup difference in the levels of other Candida species and in the levels of Staphylococcus and Enterobacteria (*P*>0.05).

**Table-III:** Comparison of clinical data between the adverse pregnancy outcome and good pregnancy outcome in patients with CIC

Item	Category	Adverse pregnancy outcomes (n = 43)	Good pregnancy outcomes (n = 182)	P
Age (years)		30 (25, 30)	29 (26.75, 33)	0.719
Gravidity (times)		2 (2, 2)	2 (1, 2)	0.073
Parity (times)		1 (1,2)	1 (1, 2)	0.781
Delivery mode	vaginal delivery	14 (32.56)	82 (45.05)	0.136
	cesarean section	29 (67.44)	100 (54.95)	0.045
CIC treatment	McDonald Cervical cerclage surgery	20 (46.51)	115 (63.19)	
	Progesterone supplementation	23 (53.49)	67 (36.81)
Weeks of cervical cerclage surgery		23.46 ± 2.75	24.13 ± 2.55	0.128
Gestational diabetes	Yes	5 (11.63)	16 (8.79)	0.777^a^
	No	38 (88.37)	166 (91.21)	
Gestational hypertension	Yes	3 (6.98)	10 (5.49)	0.991^a^
	No	40 (93.02)	172 (94.51)	
History of cervical disease	Yes	12 (27.91)	35 (19.23)	0.215
	No	31 (72.09)	147 (80.77)	
Scarred uterus	Yes	6 (13.95)	20 (10.99)	0.584
	No	37 (86.05)	162 (89.01)	
*Candida*	*C. albicans*	6 (13.95)	7 (3.85)	0.028^a^
	*C. parapsilosis*	2 (4.65)	3 (1.65)	0.531^a^
	*C. tropicalis*	1 (2.33)	2 (1.10)	0.472^b^
	Other *Candida*	0 (0.00)	2 (1.10)	1.000^b^
*Staphylococcus*	*S. aureus*	0 (0.00)	2 (1.10)	1.000^b^
	*S. epidermidis*	1 (2.33)	1 (0.55)	0.346^b^
	Other *Staphylococcus*	0 (0.00)	1 (0.55)	1.000^b^
*Enterobacteriaceae*	*E. coli*	1 (2.33)	10 (5.49)	0.636^a^
	Other *Enterobacteriaceae*	1 (2.33)	4 (2.20)	1.000^a^
*Gardneralla vaginalis*		8 (18.60)	4 (2.20)	<0.001^a^
*Ureaplasma urealyticum*		17 (39.53)	33 (18.13)	0.003
*Chlamydia*		5 (11.63)	2 (1.10)	0.002^a^

a , Yates’ correction;

b , Fisher’s exact test.

### Risk factors affecting pregnancy outcomes in women with CIC:

Based on the above univariate analysis results, CIC treatment and higher levels of Candida albicans, Gardnerella vaginalis, Ureaplasma urealyticum, and Chlamydia were used as independent variables and assigned values (Table-IV). Pregnancy outcomes (1=poor outcomes, 2=good outcomes) were used as the dependent variable in the binary logistic regression model. Elevated Candida albicans, Gardnerella vaginalis, Ureaplasma urealyticum, and Chlamydia were identified as risk factors for adverse pregnancy outcomes in patients with CIC (*P*<0.05) (Table-V).

**Table-IV:** Assignment of variables.

Variable	Assignment
CIC treatment	1 = McDonald cervical cerclage surgery, 2 = progesterone supplementation
*C. albicans*	1 = Yes; 2 = no
*G. vaginalis*	1 = Yes; 2 = no
*U. urealyticum*	1 = Yes; 2 = no
*Chlamydia*	1 = Yes; 2 = no

**Table-V:** Logistic regression analysis of risk factors affecting pregnancy outcomes in pregnant women with CIC.

Variable	B	SE	Wald	P	OR	95% CI
CIC treatment	-0.788	0.409	3.716	0.054	2.200	0.987–4.903
*C. albicans*	2.281	0.673	11.483	0.001	0.102	0.027–0.382
*G. vaginalis*	2.751	0.743	13.710	<0.001	0.064	0.015–0.274
*U. urealyticum*	2.070	0.455	20.68	<0.001	0.126	0.052–0.308
*Chlamydia*	3.441	0.983	12.263	<0.001	0.032	0.005–0.220
Constant	-2.216	0.374	35.137	<0.001	9.174	

## DISCUSSION

This study showed that the diagnosis of CIC was associated with a significantly higher number of detected Ureaplasma urealyticum strains and a considerably lower number of Escherichia coli strains compared to the uncomplicated pregnancy.

As shown in the study of Farr et al.,^13^ the bacterial metabolic pathways in pregnant women are related to changes in the microbial proteome, and bacteria can affect the metabolism of pregnant women through several pathways. Escherichia coli is an important component of normal intestinal and vaginal flora,^14^ while Ureaplasma urealyticum is a common sexually transmitted pathogen of the human urinary and reproductive tract.^15^ Under normal circumstances, acidic vaginal conditions, facilitated by vaginal lactobacilli and estrogen, effectively inhibit bacterial growth and maintain a relatively clean vaginal environment.^16^

However, CIC-associated defects in the cervical canal and relaxation of the cervical opening allow easy access of bacteria into the uterine cavity through the vagina, thereby increasing the risk of infection.^17^,^18^ A study by Xiao et al.^19^ showed that vaginal microenvironment can affect the carrier rate of Group B Streptococcus (GBS) in the reproductive tract of women in the late stages of gestation. Additionally, vulvovaginal candidiasis (VVC) and trichomonas (TV) were associated with an increased infection rate of GBS that correlated with a significant difference in the proportion of lactobacilli, a substantial increase in white blood cell count and inflammation. In this study, patients with CIC had markedly higher rates of adverse pregnancy outcomes. It is plausible that the imbalanced vaginal microbiota of CIC patients may further affect their cervical function and increase the risk of adverse pregnancy outcomes.^17^–^19^

This study identified differences in the distribution of the vaginal microbiota among CIC patients with different pregnancy outcomes. Adverse pregnancy outcomes were associated with a higher proportion of Candida albicans, Gardnerella vaginalis, Ureaplasma urealyticum, and Chlamydia. The results of the binary logistic regression model showed that Candida albicans, Gardnerella vaginalis, Ureaplasma urealyticum, and Chlamydia are risk factors for adverse pregnancy outcomes. Our results confirm previous observations. Candida albicans is a common opportunistic pathogenic fungus that populates skin, mouth, and intestines^20^ and may lead to candidiasis as a secondary infection in immunocompromised patients, pregnant women with weakened immunity, or following the use of antibiotics.^21^,^22^ Gardnerella vaginalis is a predominant anaerobic bacterium responsible for causing bacterial vaginosis and linked to complications during pregnancy.^23^

The study by Wong et al.^22^ showed a significant inflammatory response in the placenta of Gardnerella-positive pregnant women and a considerably increased number of maternal placental villous endothelial cells. Ureaplasma urealyticum was shown to colonize the human genital tract and has been associated with adverse pregnancy outcomes.^24^ Studies have shown that infection with Ureaplasma urealyticum may cause a series of reproductive tract inflammatory responses, including urethritis, cystitis, endometritis, etc.^24^,^25^ Chlamydia is a non-motile gram-negative pathogen that can cause infections in multiple organs, such as the urethra, lungs, bronchi, gastrointestinal tract, etc. The research shows that chlamydia infection in the vagina and cervix is linked to premature births and miscarriages.^26^ In a study by Van et al.^27^, Chlamydia trachomatis induced inflammation in female fallopian tubes by regulating the abnormal expression of matrix metalloproteinases (MMPs), further leading to adverse pregnancy outcomes.

The proportion of conservative treatment in the group of CIC women with poor pregnancy outcomes was higher than that in the group with good pregnancy outcomes, further confirming that emergency cerclage plays a vital role in treating cervical insufficiency and can improve pregnancy outcomes.^28^ This advantage is mainly due to the mechanical support of the cervix during surgery, which prolongs pregnancy time and provides ample space for fetal growth and development. Emergency cerclage can also help reduce the rates of miscarriages and premature births caused by cervical incompetence.^28^

However, the multivariate logistic regression analysis in our study did not identify CIC treatment as a significant factor influencing adverse pregnancy outcomes. This may be due to the presence of other contributing factors that have a more substantial impact on pregnancy outcomes than the treatment option. Previous studies showed that factors such as age, gestational age, delivery method, genetic factors, and hormone levels may affect the structure and composition of the reproductive tract microbiota and pregnancy outcomes in pregnant women with CIC.^29^,^30^ However, no such correlation was identified in this study. This discrepancy may be due to the relatively small sample size of the study.

This study has clear clinical implications. It provides further evidence of the importance of analyzing vaginal microbiota in pregnancy. Timely detection of microbiota changes can identify women who are at high risk of developing CIC and will allow clinicians to promptly initiate measures to prevent adverse pregnancy outcomes.

### Limitations:

It is a retrospective study with a relatively small sample size, which limits the generalizability of the results. Additionally, existing studies have employed various methods and standards to analyze the characteristics of microbial communities, which makes it challenging to compare and integrate their results. Therefore, further prospective studies with larger sample sizes and standardized analysis methods are necessary to accurately evaluate changes in microbial characteristics. Additionally, the distribution of vaginal microbiota in pregnant women with cervical incompetence may vary among different regions and populations. Therefore, multi-center and large-sample studies encompassing different geographic locations should further verify the universality and reliability of the results.

## CONCLUSION

The vaginal microbiota of pregnant women with CIC is diverse and is dominated by the Ureaplasma urealyticum. Higher levels of Candida albicans, Gardnerella vaginalis, Ureaplasma urealyticum, and Chlamydia are risk factors for adverse pregnancy outcomes in women with CIC. Further multi-center large-scale studies are needed to provide more convincing evidence for clinical practice.

### Authors’ contributions:

**XZ, YC, KZ, XY and JZ:** Study design, literature search, and manuscript writing.

**YC, KZ, XY and JZ:** Data collection, data analysis and interpretation. Critical review.

**XZ:** Manuscript revision and validation.

All authors have read, approved the final manuscript. They are also accountable for the integrity of the study
